# Effect of Soaking and Roasting on the Physicochemical and Pasting Properties of Soybean Flour

**DOI:** 10.3390/foods6020012

**Published:** 2017-02-09

**Authors:** Aurelie Solange Ntso Agume, Nicolas Yanou Njintang, Carl Moses F. Mbofung

**Affiliations:** 1 Department of Food Sciences and Quality Control, Institut Universitaire de Technologie (IUT), University of Ngaoundere, P.O. Box 455, Ngaoundere, Cameroon; aurelie_agume@yahoo.fr; 2 Department of Biological Sciences, Faculty of Science, University of Ngaoundere, P.O. Box 454, Ngaoundere, Cameroon; 3 Department of Food Sciences and Nutrition, ENSAI, University of Ngaoundere, P.O. Box 455, Ngaoundere, Cameroon; cmbofung@yahoo.com

**Keywords:** soybean flour, soaking, roasting, physicochemical properties, viscosity

## Abstract

The effects of soaking and roasting on the physicochemical and pasting properties of soybean flour were evaluated. Soybean seeds were soaked overnight in tap water for 0–72 h, hand dehulled, dried, and part of the sample was roasted. Roasted and unroasted soy beans were milled into flour and analyzed. The results showed that the total carbohydrates (22.8–27.9 g/100 g), the ash content (3.5–3.6 g/100 g), and the total polyphenols (0.29–0.51 g/100 g) did not significantly change during both the soaking and roasting processes. However, the total proteins (35.8–46.0 g/100 g) and lipid contents (21.4–29.5 g/100 g) were significantly (*p* < 0.05) affected only by soaking, with a decrease in total protein and an increase in lipid contents. Phytate content (0.22–0.26 g/100 g) decreased significantly (*p* < 0.05) only with roasting. The tannins (0.01–0.30 g/100 g) and soluble proteins (4.0–29.0 g/100 g) significantly (*p* < 0.05) diminished with both treatments. There was a significant increase in the least gelation concentration (20–30 g/100 mL), a decrease in the swelling power (1.3–2.0 mL/mL), and consequently, reduction in the viscosity (range peak viscosity 18–210 cP) of the flour slurry after soaking and roasting. All these qualities—needed for producing nutritious flour for infants—highlighted the efficiency of these endogenous technologies.

## 1. Introduction

Soybean is an important source of proteins (40%), lipids (20%), minerals (5%), and B vitamins for human nutrition [1]. There is increasing evidence that the consumption of soybean products reduces cancer, blood serum cholesterol, osteoporosis, chronic renal disease, heart disease, oxidative stress, and others [1,2,3]. The health benefits of soybean products have induced an increase in the demand for beans. In this respect, the world production of soybeans has significantly increased in the last decade, rising from 200 million metric tons in 2005 to 324 million metric tons in 2016 [4]. Though the 2015 annual production is very low (less than 3 million metric tons) in Africa, in this area of the world, soybean plays an important role in infant nutrition. In particular, soy flour is used to fortify traditional cereal-based foods [5,6]. Soybean-fortified maize has been the subject of biochemical, nutritional, sensory, rheological [7,8], and storage investigations. Egounlety and Syarief reported that the addition of 25% soybean tempe powder to Ogi (a fermented maize porridge) overcame the tryptophan and lysine deficiency in maize, and greatly improved the protein biological value of Ogi, without affecting the organoleptic properties of the porridge [9].

The major challenges in using soybean flour in infant food are the elimination of anti-nutrients, oligosaccharides, and beany flavor, and the reduction of the viscosity of the resulting porridge. Soaking and roasting have been applied to meet these objectives [10,11]. Roasting for 20 min at 100 °C was reported to inhibit 90% of trypsin inhibitors activity in soybean flour. It has been reported that the application of roasting achieved a pleasant flavor in soy bean [12]. Baik and Han reported a significant reduction of raffinose, verbascose, and stachyose during fermentation and roasting of soybean [11]. They equally reported that roasting induced a greater increase in protein digestibility of soybean than fermentation. Shin et al. reported increased antioxidant activity and in vitro protein digestibility in roasted soy flour [13]. In traditional households, the beans are soaked for 1–3 days, during which some microbial activities are activated, leading to improvement of the nutritional quality of the resulting flour. Recent investigations revealed a positive effect of long-time soaking in reducing the anti-nutrients and the viscosity of maize flour, but this varied with soaking time [14]. In addition, there was a significant interaction of soaking and roasting on the nutritional and pasting properties of maize flour [14]. However, this has not yet been investigated in soybean flour.

This study aims to determine the interaction effect of soaking and roasting on some physicochemical and pasting properties of soybean flour.

## 2. Materials and Methods

### 2.1. Sampling of Soybean and Production of Soybean Flour

**Figure 1:**
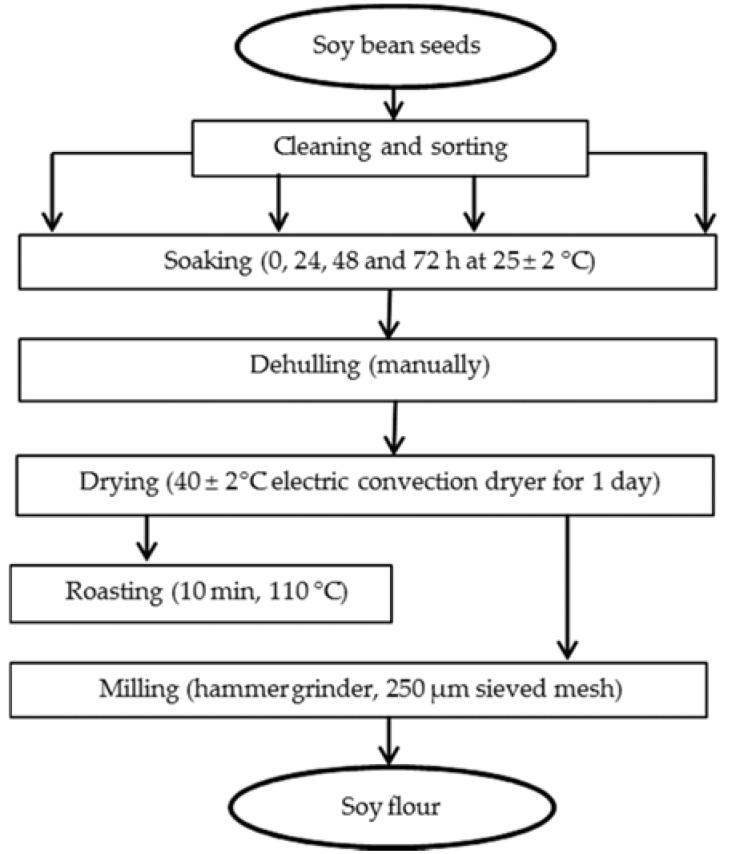
Flow diagram for the production of soybean flour.

The soybean seeds used in this study were purchased from local markets in Ngaoundere, Cameroon. The dried seeds (moisture content ≈ 11%) were carefully cleaned and sorted out to remove defective and small-sized seeds so as to obtain clean seeds of uniform size. The cleaned seeds were then processed into flour, as shown in Figure 1. The seed sample was divided into four subsamples, which were steeped in tap water (2 kg/8 L) at ambient temperature (25 ± 4 °C) for 0, 24, 48, or 72 h. Soaked seeds were manually dehulled, spread in a single layer on aluminum racks, and dried at an average temperature of 40 ± 2 °C for 1 day in a gas dryer. The dehulled subsamples were each further divided into two subsamples, from which one was roasted at 110 °C for 10 min, and the second was left unroasted. Each of the resulting eight subsamples was then milled into fine flour using a hammer electric grinder (Culatti, Polymix, France) equipped with a sieve of diameter 250 µm meshes. Flours so produced were sealed in polyethylene bags and stored at 4 °C until required for analysis.

### 2.2. Chemical Analysis of Soybean Flours

#### 2.2.1. Determination of the Proximate Composition and Some Phytochemicals

Association of Official Analytical Chemists (AOAC) methods were used to determine the moisture, crude fat, and ash contents of the flours [15]. Moisture content was determined by the direct oven drying method on 1 g of sample at 105 °C for 24 h to constant weight. Ash was determined as the residue of incinerated flour (15 g) in a crucible at 550 °C; crude fat was determined by using the Soxhlet extraction method, with hexane as solvent. Total protein (N × 6.25) was analyzed by the Kjeldahl method [16], while total carbohydrate was determined by spectrophotometric method using phenol after digestion in concentrated sulfuric acid [17].

Phytic acid, total phenolic and tannin contents were determined as recently reported [18]. Phytic acid was extracted in 1.2% HCl solution containing 10% Na_2_SO_4_ and quantified based on the formation of complex with Fe(III) ion at pH 1–2. Phenols were extracted in 80% ethanol followed by colorimetric quantification either directly in the extract (for total polyphenol determination) or after precipitation of tannins using polyvinylpyrrolidone (for non-tannin polyphenol determination). The tannin content of the sample was calculated as difference between total polyphenol and non-tannin polyphenol contents in the extract. Total phenolic and tannin contents were expressed as gallic acid and tannic acid equivalent, respectively.

#### 2.2.2. Determination of Some Physicochemical and Pasting Properties

The water absorption capacity (WAC) was determined at ambient temperature following the method described by Kaur and Singh [19]. The oil absorption capacity (OAC) expressed as grams of peanut oil per grams of flour was determined on 2.5 g sample per 30 mL oil, as described by Kaur and Singh [19]. The Least Gelation Concentration (LGC) was determined according to the method of Coffman and Garcia [20]. The swelling power was determined following the standard method reported by Gujral and Rosell [21]. The pasting properties of soybean flours were analyzed on a Rapid Visco Analyzer instrument (Tec Master Model, Perten Instruments, and Australia) as recently reported [14]. Parameters recorded were peak viscosity (PV), hold viscosity (HV: minimum viscosity at 95 °C), final viscosity (FV: viscosity at 50 °C), break down viscosity (PV–HV), and setback viscosity (FV–HV).

### 2.3. Statistical Analysis

Data were expressed as mean ± standard deviation. Two-way analysis of variance with interaction was used to determine the effect of soaking and roasting on the properties. Where the effect of a factor was significant, Duncan Multiple Range test was carried out to further determine the differences between two means. The statistical analysis of the obtained data was carried out using the Statgraphics Centurion XVI version 16.1.18 statistical package.

## 3. Results

### 3.1. Proximate Composition and Some Phytochemicals of Soybean Flour

Table 1 shows the effect of soaking time and roasting on the proximate composition of soybean flour. Generally, soybean flour is a source of lipids and proteins, and the values fall within the range reported earlier [1,11]. Similarly, the carbohydrate and ash contents are quite similar to reported values [8]. For the effect of treatments on the moisture, ash, starch, and protein contents of soybean flours, soaking and roasting induced significant changes in most cases.

Moisture content varied from 5.1 g/100 g in roasted soybean soaked for 72 h to 8.9 g/100 g in unroasted and unsoaked soybean. The moisture content did not vary significantly with soaking time. Meanwhile, roasting the soybean led to a significant (*p* < 0.02) decrease of moisture content, from a mean value of 7.2 to 5.7 g/100 g. A similar effect of soaking and roasting on the moisture content of maize flour has been reported [14], suggesting the behavior does not vary with the substrate. Since the moisture content of the flour is a consequence of its hygroscopic character, it is likely to conclude that roasting decreases the ability of soybean flour to interact with water. The low level of moisture in roasted flours probably results from the high temperature (which eliminates water more quickly), and the intermolecular cross-linking that might occur.

The total protein and carbohydrate contents ranged from 35.5 ± 1.1–46.0 ± 2.1 g/100 g dry weight (dw) and 22.8 ± 1.6–27.9 ± 1.8 g/100 g dw, respectively. The total carbohydrate content did not vary significantly with soaking or roasting. However, only soaking significantly influenced the protein content (*p* < 0.04), with soaked soybean flours generally exhibiting lower protein content than raw ones. Loss of soluble proteins during soaking probably contributed to the decrease in protein content of soybean flour. By soaking and subsequent roasting, total protein content was reduced by 23%. Decrease in proteins and total sugars during soaking and roasting of maize has been reported [14,22,23]. However, Baik and Han reported a lower (1%–7%) increase in the protein and starch of roasted and fermented soybean [12]. The difference is probably due to the fermentation process, which was a solid state fermentation with a fungus strain *Rhizopus oligosporus.* In this study, leaching induced loss of soluble nutrients, and the natural fermentation involving multiple microorganisms with variable metabolisms could have contributed to the decrease in proteins [23,24]. It is increasingly evident from studies that the drop in protein level increased with soaking time. The reduction in proteins during soaking varied from 5% for 24 h soaking to 22% for 72 h soaking.

The crude lipid content in the soybean flour samples varied between 21.4 ± 0.4 g/100 g dw (roasted-unsoaked) and 29.5 ± 1.2 g/100 g dw (roasted-72 h soaked). Only soaking induced a significant increase in the lipid content of soybean flour (*p* < 0.05). Generally, the crude lipid content of soybean flour increased with soaking by 20%–38% in roasted soybean flour and 13%–20% in unroasted soybean flour. This probably resulted as a consequence of the leaching of soluble components, causing a concentration of the lipids in the flour. Meanwhile, roasting the seeds induced a non-significant increase in the lipid content of the soybean flour by 0%–11%. An increase in oil content after roasting has been reported for cereal seeds including millet [25], maize [26], and sesame [27]. The increase in the crude fat content may result from the destruction of cell structure and the efficient release of oil reserve [28].

The ash content of soybean flour varied from 3.48–3.55 g/100 g dw and from 3.47–3.59 g/100 g dw for roasted and unroasted soybeans, respectively. The low values observed in this study as compared to the 6.0% previously reported [8] might be a result of soaking (which solubilized minerals), or the difference in variety and agro-ecological zone of cultivation. Soaking time and roasting did not cause a significant difference in the ash content of the soybean flour.

**Figure 2:**
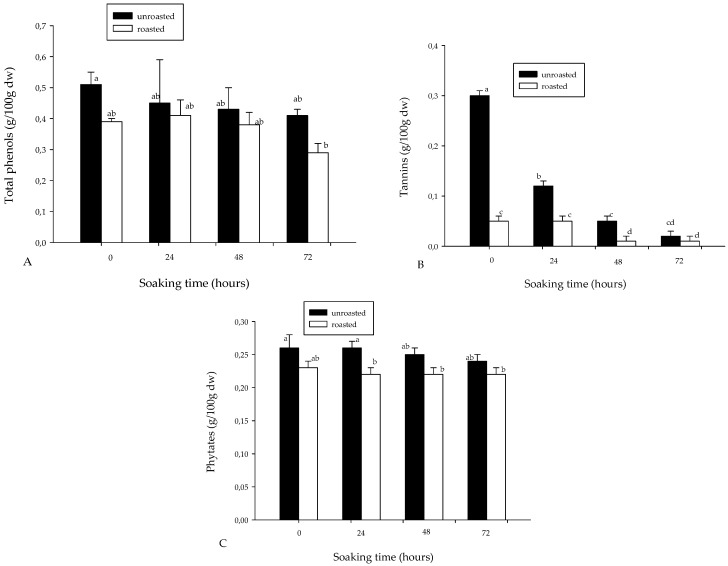
Total (**A**) polyphenol; (**B**) tannin; and (**C**) phytate contents of soybean flour as affected by soaking time and roasting. Bars bearing different letters are significantly different at *p* < 0.05; *n* = 3. DW: dry weight.

Figure 2 shows the levels in some phytochemicals of soybean flour. The total polyphenol content (0.29–0.51 g/100 g dw) did not vary significantly with soaking and roasting (Figure 2A). However, tannin (0.01–0.30 g/100 g dw) content of the soybean flour decreased generally, not only with soaking, but also with roasting (Figure 2B). In similar conditions during 48 h soaking and roasting of maize, there was a 22% decrease [14]. The decrease in tannins may result from leaching into the soaking water. In the past, polyphenols and tannins were considered as anti-nutrients because they can interfere and precipitate proteins, thus reducing their biological utilization. In this respect, the decrease in tannins can be considered as positive. However, in recent years, they have been given positive consideration as dietary antioxidants and health-promoting phytochemicals [26]. In this respect, soybean polyphenols—in particular, the isoflanones such as genistin, a biologically-active oestrogen-like compound—have been associated with the antioxidant activity of soybean flour [29,30]. It was expected that fermentation would occur as a result of soaking the soybean seeds, as the polyphenol content and antioxidant activity of soybean flour has been shown to increase through the catalytic action of the β-glucosidase enzyme produced by the microorganisms, which hydrolyzes isoflavone glucosides (isoflavones are mainly present in soybean foods in the form of glucoside) and liberates lipophilic aglycone. In this respect, solid-state bioprocessing of soybean has been suggested to produce value-added soybean flour [30,31]. Since polyphenols may be considered positively or negatively, comparative study needs to be carried out on the antioxidant and protein biological value of the soybean flour obtained by the liquid and solid-state fermentation processes.

Figure 2C shows the variation in phytate content (0.22–0.26 g/100 g dw), which significantly (*p* < 0.01) decreased with roasting, while soaking had no significant effect. This observation is in agreement with earlier studies, which reported a significant decrease in phytate content during soaking and roasting of maize flour [14,23,25,27]. In particular, there was a 15% decrease of phytates in soybean flour during 48 h of soaking and roasting, while in similar conditions, there was an 18% decrease in the phytates of maize [14]. Contrary to this, studies by Egounlety and Aworh reported non-significant changes in phytate content during 12–14 h soaking in soybeans, cowpea, and ground bean [32].

### 3.2. Variation in Soluble Proteins and Sugars

**Figure 3:**
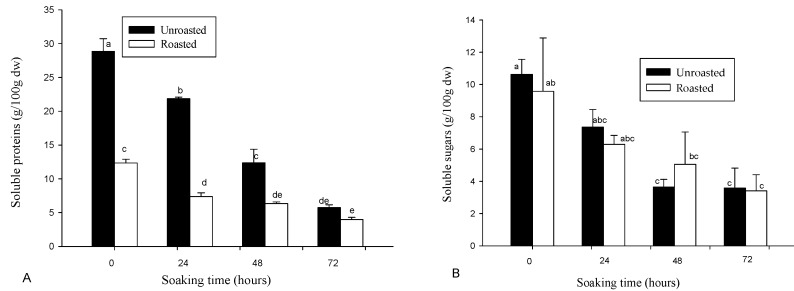
(**A**) Soluble protein; and (**B**) Soluble sugar contents of soybean flour, as affected by soaking time and roasting. Bars bearing different letters are significantly different at *p* < 0.05; *n* = 3.

The soluble protein and sugar contents of soybean flour as influenced by the treatment conditions are presented in Figure 3. Generally, the soluble protein (4.0 ± 0.3–29.0 ± 1.9 g/100 g dw) content decreased significantly (*p* < 0.001) with both soaking and roasting. In addition, the soluble sugar content (3.4 ± 1.0–10.6 ± 0.9 g/100 g dw) decreased significantly (*p* < 0.01) with the soaking time, while roasting had no significant effect. Roasting induced a 33% to 66% decrease in soluble protein, while the reduction due to soaking ranged 64%–66% for soluble sugars and 67%–80% for soluble proteins. Egounlety and Aworh reported the major oligosaccharides in soybean as sucrose, raffinose, and stachyose, with respective contents of 4.91 g/100 g dw, 1.22 g/100 g dw, and 8.41 g/100 g dw [32]. They reported a 20%–26% decrease in each of the oligosaccharides during 12–14 h soaking of the soybean seeds. A similar decrease (16%–34%) was reported in cowpea and ground bean [32]. The more important decrease observed in this study is probably a result of long soaking duration, which could have induced a natural fermentation, as demonstrated earlier [14]. In this respect, Egounlety and Aworh reported 56.8% and 10% decreases in stachyose and raffinose, respectively, during 48 h solid state fermentation with *Rhizopus*, while verbascose disappeared totally [32]. In the same vein, reductions of 87% and 53% in soybean soluble oligosaccharides have been reported during fermentation and roasting, respectively [11]. The same group also reported in both conditions, respectively, 35% and 96% reductions of soluble soybean proteins [11]. Similar reductions in soluble oligosaccharides and proteins following roasting and fermentation were equally observed in other dry legume seeds, such as chickpeas, lentils, and peas [11]. The reduction in soluble sugars and proteins during fermentation is probably a consequence of their use for the growing microorganisms. Several other authors reported an increase in soluble proteins and sugars during the fermentation of cereals as a consequence of depolymerisation through enzymatic hydrolysis [33]. The difference in the behavior is probably a consequence of the difference in the composition of cereals and legumes. Legumes are rich sources of soluble oligosaccharides and proteins as compared to cereals [14], and in this respect, do not need the initial hydrolysis of starch and proteins to initiate growth.

### 3.3. Some Physicochemical and Pasting Properties of Soybean Flour

**Figure 4:**
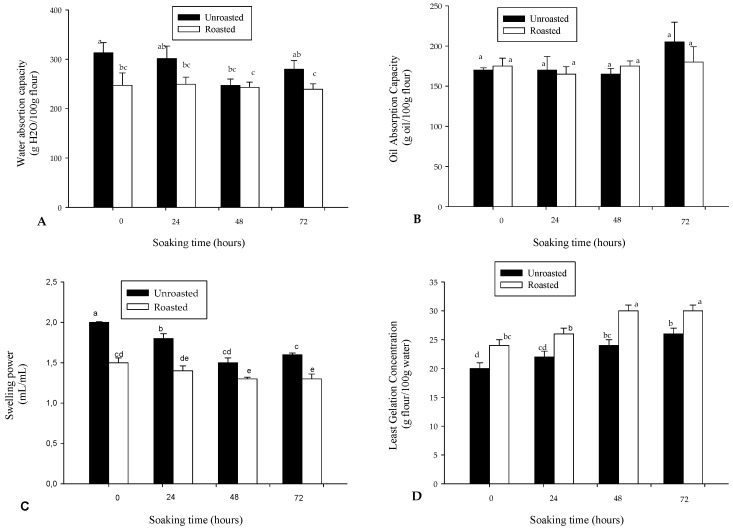
Variation in (**A**) Water absorption capacity; (**B**) Oil absorption capacity; (**C**) Swelling power; and (**D**) Least gelation concentration of roasted and unroasted soybean flour as a function of soaking time. Bars bearing different letters are significantly different at *p* < 0.05; *n* = 3.

The Water absorption capacity (WAC) of soybean flour is presented in Figure 4A. The WAC of soybean flour ranged 239–313 g/100 g, and decreased significantly (*p* < 0.01) with only roasting. Roasting induced a 2% to 23% decrease in the WAC of soybean flour. These observations contrasted with some reports which showed either a non-significant change for maize [14] or an increase for dry legumes [11], but are similar to those reported from other studies [34,35]. The soaking and roasting type might have influenced the water absorption behavior. Values of WAC observed in the present work are higher (213%) than values reported for soybean flour elsewhere [8]. In addition, the WAC reported for soybean flour is systematically higher than that reported for maize and other cereal flour (92–151 g/100 g) [8,14].

Compared to water absorption capacity, the oil absorption capacity (OAC) (Figure 4B) did not change significantly with either soaking or roasting. In addition, the ability of soybean flour to bind and retain oil (OAC range 165 ± 7–205 ± 24 g oil/100 g) was lower as compared to the ability to bind and retain water (WAC range 239–313 g/100 g flour). Akubor and Animawo reported similar observations for soybean [8]. However, the swelling (Figure 4C) significantly (*p* < 0.001) diminished with soaking time and roasting. The decrease in swelling with soaking time was 10%–25% and 6%–13% for unroasted and roasted soybean flour, respectively. As for WAC, the swelling decreased with roasting by 13%–25%, and in general, 48 h soaking associated with roasting induced a 35% reduction in swelling power. The range of swelling values were 1.3–2.0 mL/mL—higher than that reported for maize flour [14]. Swelling is a characteristic mostly attributed to starch, but which interacts with other components such as proteins and lipids. The reduction of swelling might result from the destruction of starch granules and proteins’ structure through microorganism-induced enzymatic hydrolysis of peptide and glycosidic bonds.

Soaking and roasting are important traditional technologies applied to cereals and dry seeds for the production of infant foods. In this respect, gelation is a negative behavior needed for the flour. The least gelation concentration (LGC, which expresses the quantity of flour needed per volume to obtain a gel) is presented in Figure 4D. The higher the LGC, the higher the potential to produce flour with high energy density. It was observed that the LGC increased significantly (*p* < 0.001) with soaking and roasting. While raw soybean flour had an LGC of 20 g/100 mL, the 72 h soaked and roasted soybean flour exhibited an LGC of 30 g/100 mL, equivalent to a 50% increase. A similar increase after 48–72 h soaking, although lower, was reported in cereal flours [14,25].

**Figure 5:**
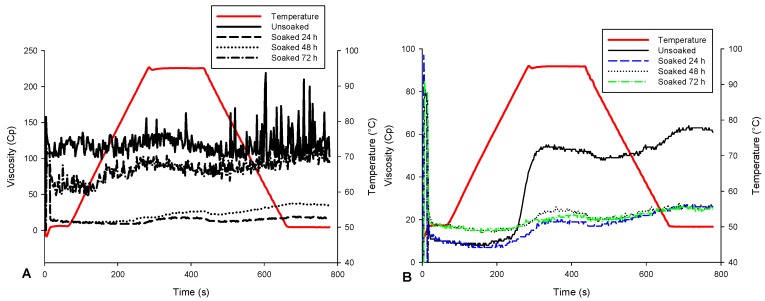
Viscoamylograph profiles of (**A**) unroasted; and (**B**) roasted soaked and unsoaked soybean flour.

The most important parameter to appreciate the increase in the energy density of the flour during treatment is the viscosity. Generally, flours with high gelling power exhibited high swelling ability, and the resulting slurry had high viscosity. The viscoamylographs of the soybean flour are presented in Figure 5. The change in the viscosity of soybean slurry during heating showed a behavior uncharacteristic of starch solution. In particular, the viscosity of unroasted soybean slurry did not change significantly during heating, probably as a consequence of the interaction of starch with lipid and its low starch content. In addition, the viscosity diminished significantly (*p* < 0.05) with soaking, and no difference can be visualized between curves for 24, 48, and 72 h soaking. Similar observations were made for the viscosity of roasted soybean slurry. However, the behavior of roasted unsoaked soybean was typical to that of starch, with a clearly identified pasting point and peak viscosity. The pasting parameters of the soybean flours are reported in Table 2, and it is evident that roasting of unsoaked seeds led to a significant (*p* < 0.05) decrease in the peak (74%), final (41%), hold (47%), and breakdown (94%) viscosities of the soybean flour slurry. Similar reductions of viscosity was reported in maize [14] and a blend of maize–soybean flour [36].

## 4. Conclusions

This study reveals that the effect of soaking and roasting depends on the property studied. While soaking induced a decrease in total protein, soluble sugar and tannins, and an increase in lipid content, roasting led to a decrease in the tannin, phytate, and soluble protein contents of the soybean flour. In addition, the swelling index, gelling ability, and viscosity of the soybean flour diminished considerably with soaking and roasting, while the ability to bind and retain water only decreased with roasting. The reductions in these properties are more important when soaking is coupled with roasting. This study highlights the positive effect of endogenous technologies on the improvement of energy density of soy bean flour slurry. However, the nutritional and sensory properties and the microbiological quality of the foods made from soaked roasted soybean flour need to be investigated.

## Figures and Tables

**Table 1:** Proximate composition (expressed in g/100 g dry weight basis) of soybean flour as affected by roasting and soaking time.

Parameters (%)	Roasted Soybean Flour				Unroasted Soybean Flour			
	Unsoaked	Soaked 24 h	Soaked 48 h	Soaked 72 h	Unsoaked	Soaked 24 h	Soaked 48 h	Soaked 72 h
Moisture *	6.9 ± 0.5 ^a,b^	5.4 ± 0.5 ^b^	5.3 ± 0.3 ^b^	5.1 ± 0.5 ^b^	8.8 ± 1.6 ^a^	6.8 ± 0.3 ^a^	6.9 ± 0.4 ^a^	6.7 ± 0.4 ^a^
Total carbohydrates	27.0 ± 1.1 ^a^	26.9 ± 1.8 ^a^	25.1 ± 1.4 ^a^	24.3 ± 1.8 ^a^	27.8 ± 1.8 ^a^	22.8 ± 1.6 ^a^	27.9 ± 1.8 ^a^	24.2 ± 1.3 ^a^
Total proteins	44.1 ± 2.1 ^a,b^	35.8 ± 3.3 ^b^	37.0 ± 2.3 ^a,b^	35.5 ± 1.1 ^b^	46.0 ± 2.1 ^a^	43.9 ± 3.3 ^a,b^	37.9 ± 4.3 ^a,b^	35.8 ± 3.3 ^b^
Crude lipids	21.4 ± 1.4 ^c^	25.7 ± 1.3 ^a,b,c^	28.0 ± 1.6 ^a,b^	29.5 ± 1.2 ^a^	22.6 ± 1.1 ^c^	25.5 ± 1.5 ^a,b,c^	25.1 ± 1.8 ^b,c^	27.2 ± 1.2 ^a,b^
Ash	3.5 ± 0.2 ^a^	3.5 ± 0.1 ^a^	3.5 ± 0.1 ^a^	3.6 ± 0.1 ^a^	3.6 ± 0.1 ^a^	3.6 ± 0.1 ^a^	3.5 ± 0.1 ^a^	3.5 ± 0.1 ^a^

Mean ± standard deviation; values with different letters within the same row differ significantly (*p* ˂ 0.05) as determined by Duncan’s multiple range test (*n* = 3). * Moisture is expressed based on fresh weight.

**Table 2:** Pasting properties of soy flour as affected by roasting and soaking time.

Treatments		Parameters				
Roasting	Time of Soaking (h)	Peak Viscosity (cP)	Hold Viscosity (cP)	Final Viscosity (cP)	Breakdown Viscosity (cP)	Set back Viscosity (cP)
Unroasted						
	0	210 ± 1 ^a^	91 ± 1 ^a^	103 ± 3 ^a^	119 ± 1 ^a^	12 ± 1 ^b^
	24	18 ± 2 ^e^	12 ± 3 ^e^	18 ± 2 ^f^	6 ± 1 ^c,d^	6 ± 1 ^c^
	48	27 ± 2 ^d^	22 ± 2 ^d^	35 ± 1 ^d^	5 ± 1 ^c,d^	13 ± 1 ^b^
	72	104 ± 3 ^b^	69 ± 3 ^b^	95 ± 1 ^b^	35 ± 1 ^b^	26 ± 1 ^a^
Roasted						
	0	55 ± 4 ^c^	48 ± 2 ^c^	61 ± 2 ^c^	7 ± 1 ^c^	13 ± 1 ^b^
	24	20 ± 3 ^d,e^	17 ± 2 ^d,e^	26 ± 1 ^e^	3 ± 1 ^d^	9 ± 1 ^c^
	48	26 ± 2 ^d^	19 ± 3 ^d,e^	26 ± 1 ^e^	7 ± 1 ^c^	7 ± 1 ^c^
	72	23 ± 1 ^d^	19 ± 2 ^d^	25 ± 1 ^e^	6 ± 1 ^c,d^	6 ± 1 ^c^

Mean ± standard deviation; values with different letters within the same row differ significantly (*p* ˂ 0.05) as determined by Duncan’s multiple range test (*n* = 3).
